# Differential uptake of arginine derivatives by the human heteromeric amino acid transporter b^0,+^AT-rBAT (*SLC7A9*-*SLC3A1*)

**DOI:** 10.1007/s00210-024-03510-z

**Published:** 2024-10-21

**Authors:** Sofna Banjarnahor, Lorenz A. Scherpinski, Max Keller, Jörg König, Renke Maas

**Affiliations:** 1 Institute of Experimental and Clinical Pharmacology and Toxicology, Friedrich-Alexander-Universität Erlangen-Nürnberg, 91054 Erlangen, Germany; 2 Research Centre for Pharmaceutical Ingredient and Traditional Medicine, Cibinong Science Center, National Research and Innovation Agency (BRIN), 16911 Cibinong, Jawa Barat Indonesia; 3 FAU NeW Research Center New Bioactive Compounds, Friedrich-Alexander-Universität Erlangen-Nürnberg, 91054 Erlangen, Germany; 4 Institute of Pharmacy, Universität Regensburg, 93040 Regensburg, Germany

**Keywords:** b^0,+^AT-rBAT, *SLC7A9*, *SLC3A1*, L-arginine, L-homoarginine, ADMA, Proximal tubule, Transport

## Abstract

L-arginine and its (patho-)physiologically active derivatives, L-homoarginine and asymmetric dimethylarginine (ADMA), show significant differences in their renal clearance. The underlying molecular mechanisms remain to be elucidated, but selective tubular transport protein-mediated mechanisms likely play a role. In the present study, we investigate the human heteromeric transporter b^0,+^AT-rBAT (encoded by the *SLC7A9* and *SLC3A1* genes) as a potential candidate because it is localized in the luminal membrane of human proximal tubule cells and capable of mediating the cellular uptake of amino acids, including L-arginine. Double-transfected Madin-Darby canine kidney (MDCK) cells stably expressing human b^0,+^AT-rBAT exhibited significant uptake of L-arginine and L-homoarginine, with apparent *K*_m_ values of 512.6 and 197.0 μM, respectively. On the contrary, ADMA uptake was not saturated up to 4000 μM, with a transport rate > 5 nmol × mg protein^−1^ × min^−1^. With an IC_50_ value of 115.8 μM, L-arginine inhibited L-homoarginine uptake. Conversely, L-arginine only exhibited a partial inhibitory effect on ADMA uptake. Taken together, our data indicate that b^0,+^AT-rBAT may contribute to the differential renal handling of L-arginine, L-homoarginine, and ADMA.

## Introduction

In the kidneys, glomerular filtration is complemented by selective tubular transport processes to prevent the loss of desired compounds (Gansevoort et al. 2013) and to enhance the elimination of toxic substances (Vanholder et al. 2018). As a result, chemically related but (patho-)physiologically distinct compounds can differ substantially in their renal handling. Among others, this has been reported for L-arginine, L-homoarginine, and asymmetric dimethylarginine (ADMA) with renal clearances of approximately 69, 0.7, and 0.1 mL/min for ADMA, L-homoarginine, and L-arginine, respectively (Achan et al. 2003; Brosnan and Brosnan 2004; Cox and Cameron 1974; Doolan et al. 1955; Frenay et al. 2015; Marescau et al. 1997; Tizianello et al. 1980; van de Poll et al. 2004).

The semi-essential amino acid L-arginine is involved in various metabolic and signaling pathways. It is an important substrate for various metabolic pathways, including protein synthesis and L-arginine-nitric oxide signaling (Morris 2016). L-homoarginine is itself a substrate of enzymes in several L-arginine pathways and has been characterized as a protective risk marker and possible protective factor for cardiovascular disease and total mortality (Pilz et al. 2014; Tomaschitz et al. 2014; Zinellu et al. 2018). Conversely, numerous studies have linked asymmetric dimethylarginine (ADMA), a nitric oxide synthase (NOS) inhibitor, to adverse outcomes (Schlesinger et al. 2016).

Given the different pathophysiological roles of L-arginine, L-homoarginine, and ADMA, a better understanding of the mechanisms underlying their differential renal handling may help to understand the observed differential effects of various drugs on these metabolites (Maas et al. 2020) and to devise more selective therapeutic interventions to target pathologies associated with the presence or absence of these metabolites. To identify potential candidates for the reabsorption and differential handling of L-arginine and its derivatives, we assessed the transport proteins that have been identified or postulated for these substrates so far (Banjarnahor et al. 2020) and identified the heteromeric protein b^0,+^AT-rBAT (*SLC7A9*-*SLC3A1*) as a promising candidate.

The abbreviated name b^0,+^AT-rBAT relates to its functional properties: The “r” in rBAT is referred to as “related to BAT (BAT = b^0,+^ amino acid transport), “b” for broad, with a lowercase letter representing sodium independent active uptake, and the superscripts “0” and “ + ” designating the net charge on the neutral and cationic amino acid substrates, respectively. rBAT functions as the anchor and guiding protein, which is important for the localization, and co-expression of rBAT with b^0,+^AT leads to apical expression of the complex. b^0,+^AT-rBAT is encoded by the *SLC7A9* and *SLC3A1* genes (Bauch and Verrey 2002; Mizoguchi et al. 2001). When functioning as a complex (Wu et al. 2020), the b^0,+^AT-rBAT heteromer facilitates the influx of cationic amino acids (such as L-arginine, L-lysine, and L-ornithine) while also enabling the release of neutral amino acids from within the cell (Bauch and Verrey 2002). The rBAT protein is expressed mainly in the apical membrane of epithelial cells of the kidney and small intestine (Furriols et al. 1993). In addition, the b^0,+^AT protein is expressed explicitly in the kidney, small intestine, and placenta (Furriols et al. 1993). Even though both proteins are expressed in the renal proximal tubules, their expression shows opposing gradients along the renal tubule (Fernandez et al. 2002). This difference in expression patterns is due to *SLC3A1* having an additional light chain (Nagamori et al. 2016), which influences its spatial distribution compared to *SLC7A9* (Chairoungdua et al. 1999; Feliubadaló et al. 1999; Furriols et al. 1993; Kanai et al. 1992).

Due to its association with cystinuria, the b^0,+^AT-rBAT protein is often referred to as a “cystine transporter” (Fernandez et al. 2002). However, in patients with cystinuria, the mutations in the *SLC7A9* and *SLC3A1* genes do not only lead to cystinuria but also to argininuria and homoargininuria, indicating a crucial role in the tubular reabsorption of these substrates as well (Cox and Cameron 1974; Fjellstedt et al. 2003; Palacín 2009). A direct comparison of the transport properties of b^0,+^AT-rBAT for L-arginine, L-homoarginine, and ADMA has not been available, so far.

Therefore, this study aimed to assess and compare the apparent kinetics of b^0,+^AT-rBAT-mediated cellular uptake of L-arginine, L-homoarginine, and ADMA using a MDCK (Madin-Darby canine kidney) cell model overexpressing human b^0,+^AT-rBAT.

## Materials and methods

### Materials

Unlabeled L‐arginine and ADMA were purchased from Enzo Life Sciences GmbH (Lörrach, Germany). Unlabeled L-homoarginine was obtained from Sigma-Aldrich Chemie GmbH (Taufkirchen, Germany). Unlabeled L-cystine was obtained from Carl Roth Chemie GmbH (Germany). [^3^H]L-arginine (40 Ci/mmol) was obtained from American Radiolabeled Chemicals, Inc. (St. Louis, MO, USA). [^3^H]L-homoarginine (6 Ci/mmol) was obtained from ViTrax Co. (CA, USA). [^3^H]ADMA (25 Ci/mmol) was purchased from BIOTREND Chemikalien GmbH (Cologne, Germany). Minimum essential medium (MEM), fetal bovine serum, hygromycin B (50 mg/mL), penicillin–streptomycin solution, 0.05%-trypsin–EDTA solution, the Pierce BCA Protein Assay Kit, and SYTOX Green nucleic acid stain were obtained from Thermo Fisher Scientific (Dreieich, Germany). Geneticin (G418), vectors pCR2.1-TOPO, pcDNA3.1( −), and pcDNA3.1( +) were obtained from Invitrogen GmbH (Karlsruhe, Germany). ﻿Complete Mini Protease Inhibitor Cocktail Tablets and the LightCycler® FastStart DNA Master^PLUS^ SYBR Green I Kit were obtained from Roche Diagnostics GmbH (Mannheim, Germany). A QuikChange Lightning Multi-site-directed mutagenesis kit was purchased from Agilent Technologies Deutschland GmbH (Waldbronn, Germany). The Effectene® transfection reagent was obtained from Qiagen (Hilden, Germany). Cell culture flasks (25/75 cm^2^) were obtained from ﻿Sarstedt AG & Co. (Nümbrecht, Germany). CELLSTAR® 12-well cell culture Multiwell Plate and ﻿ThinCert™ cell culture inserts (PET 0.4 µm) were obtained from Greiner Bio-One GmbH (Frickenhausen, Germany). The NucleoSpin® RNA Plus Kit was obtained from MACHEREY–NAGEL GmbH & Co. (Düren, Germany). The iScript™ cDNA Synthesis Kit was obtained from Bio-Rad Laboratories GmbH (München, Germany). Unless specified otherwise, all other chemicals and reagents were obtained from Carl Roth GmbH & Co. KG (Karlsruhe, Germany) and were of the highest grade available.

### Antibodies

The rabbit anti-SLC7A9 polyclonal antibody (RRID: AB_10857796) was obtained from Biozol (Eching, Germany). The mouse anti-SLC3A1 monoclonal antibody was obtained from Santa Cruz Biotech (Heidelberg, Germany). A mouse anti-β-actin monoclonal antibody (RRID: AB_476743) was purchased from Sigma-Aldrich (St. Louis, USA). The goat anti-rabbit IgG horseradish peroxidase-labeled antibody (RRID: AB_2650489) was obtained from GE Healthcare Life Sciences (Buckinghamshire, UK). Horseradish peroxidase-labeled goat anti-mouse IgG (RRID: AB_2491007) was obtained from Dianova GmbH (Hamburg, Germany). The goat anti-mouse IgG Alexa Fluor Plus 647 antibody (RRID: AB_2633277) and the Alexa Fluor 568 goat anti-rabbit IgG antibody (RRID: AB_143157) were obtained from Thermo Fisher Scientific (Dreieich, Germany).

### Cell culture

Parental MDCKII and MDCKII control (MDCK-VC) cell lines were obtained from the Institute of Experimental and Clinical Pharmacology and Toxicology at Friedrich-Alexander-Universität Erlangen-Nürnberg. MDCKII cells were used to generate stable transfectants. All cell lines were cultured in minimum essential medium (MEM) supplemented with 10% fetal bovine serum, 100 U/mL penicillin, and 100 μg/mL streptomycin at 37 °C and 5% CO_2_. Hygromycin B (250 μg/mL) or geneticin (800 μg/mL) was added to the media for selective cell cultivation.

### Cloning of the *SLC7A9* and *SLC3A1* cDNAs encoding human b^0,+^AT and rBAT, respectively

The cDNAs encoding human b^0,+^AT (*SLC7A9*) and human rBAT (*SLC3A1*) were cloned using an RT-PCR-based approach with the following PCR primers: hb^0,+^AT: forward (5′-GGA ACC AGC AGG AGG AAA CAT G-3′) and reverse (5′-GAC GGA GCT TGT TAC TCA GGG T-3′); hrBAT: forward (5′-GTC GGT GAG ACA TGG CTG AAG AT-3′) and reverse (5′- GGT GCC TAA CAC GAG GTA TAC AG-3′). Total human kidney RNA (purchased from Takara Bio Europe, Saint-Germain-en-Laye, France) was used as template for RT-PCR. The amplified *SLC7A9* cDNA was first cloned into the cloning vector pcR2.1-Topo and finally subcloned and inserted into the vector pcDNA3.1/Hygro( −), resulting in the vector pb^0,+^AT.31, whereas the *SLC3A1* cDNA was finally subcloned and inserted into the expression vector pcDNA3.1/Geneticin( +) to establish the vector prBAT.31. Single nucleotide variations identified by sequencing compared to the reference sequences NM_001126335 (b^0,+^AT) and NM_000341.3 (rBAT) were corrected using the QuikChange Multi Site-Directed Mutagenesis Kit.

### Generation of stably transfected cells

Using the Effectene® Transfection Reagent Kit (Qiagen GmbH, Hilden, Germany), MDCKII cells were transfected with the expression vector pb^0,+^AT.31 containing *SLC7A9* cDNA. After treatment with hygromycin B (250 μg/mL), single colonies (MDCK-b^0,+^AT) were selected and screened for *SLC7A9* mRNA expression. The cell clone with the highest mRNA expression was then transfected with the vector prBAT.31, and clones were selected using the same procedure.

### RNA isolation and quantitative RT-polymerase chain reaction

Total RNA was isolated from MDCK-b^0,+^AT-rBAT double-transfected and MDCK-b^0,+^AT single-transfected cells with a NucleoSpin® RNA Plus Kit according to the manufacturer’s instructions. For each sample, 1 µg of total RNA was used for cDNA synthesis using the iScript™ cDNA Synthesis Kit. ﻿Quantitative PCR was performed using LightCycler® FastStart DNA Master^PLUS^ SYBR Green I reagents (Roche Diagnostics-Applied Science) and the following primers: hb^0,+^AT: forward (5′-TGG AAA GGC CTA TCA AGG TGCC-′3) and reverse (5′-GAC GGA GCT TGT TAC TCA GGG T-3′); hrBAT: forward (5′-GTT GAT GTC CAA AAG ACT CAG CCC-3′) and reverse (5′-GGT GCC TAA CAC GAG GTA TAC AG-3′); and β-actin (*ACTB*): forward (5′-﻿TGA CGG GGT CAC CCA CAC TGT GCC CAT CTA-3′) and reverse (﻿5′-CTA GAA GCA TTT GCG GTG GAC GAT GGA GGG-3′). The expression of each mRNA was calculated via linear regression and normalized to the expression of the housekeeping gene β-actin (*ACTB*).

### Immunofluorescence microscopy

Cells were seeded on tissue culture (TC) inserts (PET 0.4 µm, Greiner Bio-One, Frickenhausen, Germany) and cultivated for 3 days to 100% confluency. Sodium butyrate was added to the culture medium 24 h before the experiment. The filters were washed three times with PBS. The cells were fixed using 70% ice-cold methanol for 10 min and incubated with 0.4% Triton X-100 for 10 min at RT. Then, the filters were cut into squares and incubated with 2% bovine serum albumin (BSA) solution for 60 min at RT. b^0,+^AT-rBAT and b^0,+^AT immunofluorescence was performed with a mixture of the respective antibodies b^0,+^AT (rabbit anti-SLC7A9 polyclonal antibody; 1:500) and rBAT (mouse anti-SLC3A1 monoclonal antibody; 1:50) overnight at 4 °C. After washing with 0.05% TBS buffer and 0.1% PBS-Triton solution, the filter pieces were incubated for 6 h at RT with anti-rabbit IgG conjugated with Alexa Fluor 568 (1:2,000) and goat anti-mouse IgG conjugated with Alexa Fluor 647 (1:2,000). Nuclei were stained with SYTOX Green (1:50,000). ﻿Microscopy was performed on a Zeiss Spinning Disc Axio Observer Z1 at OICE (Erlangen, Germany).

### Uptake transport assays

MDCK-b^0,+^AT-rBAT double-transfected, MDCK-b^0,+^AT single-transfected, and MDCK-VC (control) cells were grown on tissue culture inserts (PET 0.4 µm, Greiner Bio-One, Frickenhausen, Germany) to confluence for 3 days at initial densities of 10 × 10^5^, 8 × 10^5^, and 5 × 10^5^ cells/well, respectively. Twenty-four hours before the uptake experiments, the cells were treated with sodium butyrate (at a final concentration of 10 mM) because Na-butyrate increases protein expression as a histone deacetylase inhibitor (Davie 2003). Before the uptake experiments, the cells were washed with prewarmed (37 °C) transport buffer (142 mmol/L NaCl, 5 mmol/L KCl, 1 mmol/L K_2_HPO_4_, 1.2 mmol/L MgSO_4_, 1.5 mmol/L CaCl_2_, 5 mmol/L glucose, and 12.5 mmol/L HEPES, pH 7.3) and incubated with a mixture of radiolabeled and unlabeled L-arginine, L-homoarginine, or ADMA, respectively, in transport buffer at 37 °C for the desired time points. Then, the cells were washed three times with ice-cold uptake buffer. Subsequently, the filter inserts were excised, cells were lysed with 0.2% SDS, and 400 μL of each aliquot was used to determine the intracellular accumulation of radioactivity (cpm) by liquid scintillation counting (TriCarb 2800; PerkinElmer, LAS GmbH, Rodgau-Jügesheim). Additional inserts were prepared similarly and excised, and cells were lysed with 0.2% SDS and prepared for determination of the cellular protein concentration via a BCA assay. The rate of uptake was normalized to the protein value of each well. The data were combined from at least two single experiments performed in duplicate (*n* ≥ 2 × 2 = four biological replicates) on different days.

Time-dependent experiments were conducted to determine the period of linear substrate uptake. For this purpose, the cells were incubated at 37 °C with transport buffer containing 100 μM L-arginine, 1 μM L-homoarginine, or 1 μM ADMA (all unlabeled spiked with the respective radiolabeled version) for 1, 2, 5, or 10 min. Increasing concentrations of L-arginine, L-homoarginine, and ADMA were used to determine the kinetic parameters (*K*_m_ values) of b^0,+^AT-rBAT-mediated transport. To investigate the inhibition of b^0,+^AT-rBAT-mediated uptake of L-homoarginine and ADMA by L-arginine and L-cystine, cells were co-incubated with various concentrations of the potential inhibitors. Furthermore, to confirm that the observed cellular uptake was transporter-mediated rather than passive diffusion, the uptake was also measured at an incubation temperature of 4 °C, where transporter-mediated uptake is significantly reduced.

### Statistical analysis

GraphPad Prism 9.0 was used for statistical analysis (GraphPad Software, San Diego, CA). The net uptake was calculated by subtracting the uptake into MDCK-VC cells from the uptake into MDCK-transporter-overexpressing cells. One- or two-way ANOVA followed by a Tukey or Bonferroni post hoc correction for multiple comparisons and a *t*-test were used for statistical analysis. The values are reported as the mean ± standard error of the mean (SEM).

## Results

### Analysis of single-transfected MDCK cells stably expressing b^0,+^AT and double-transfected MDCK cells expressing b^0,+^AT and rBAT

**Fig. 1:**
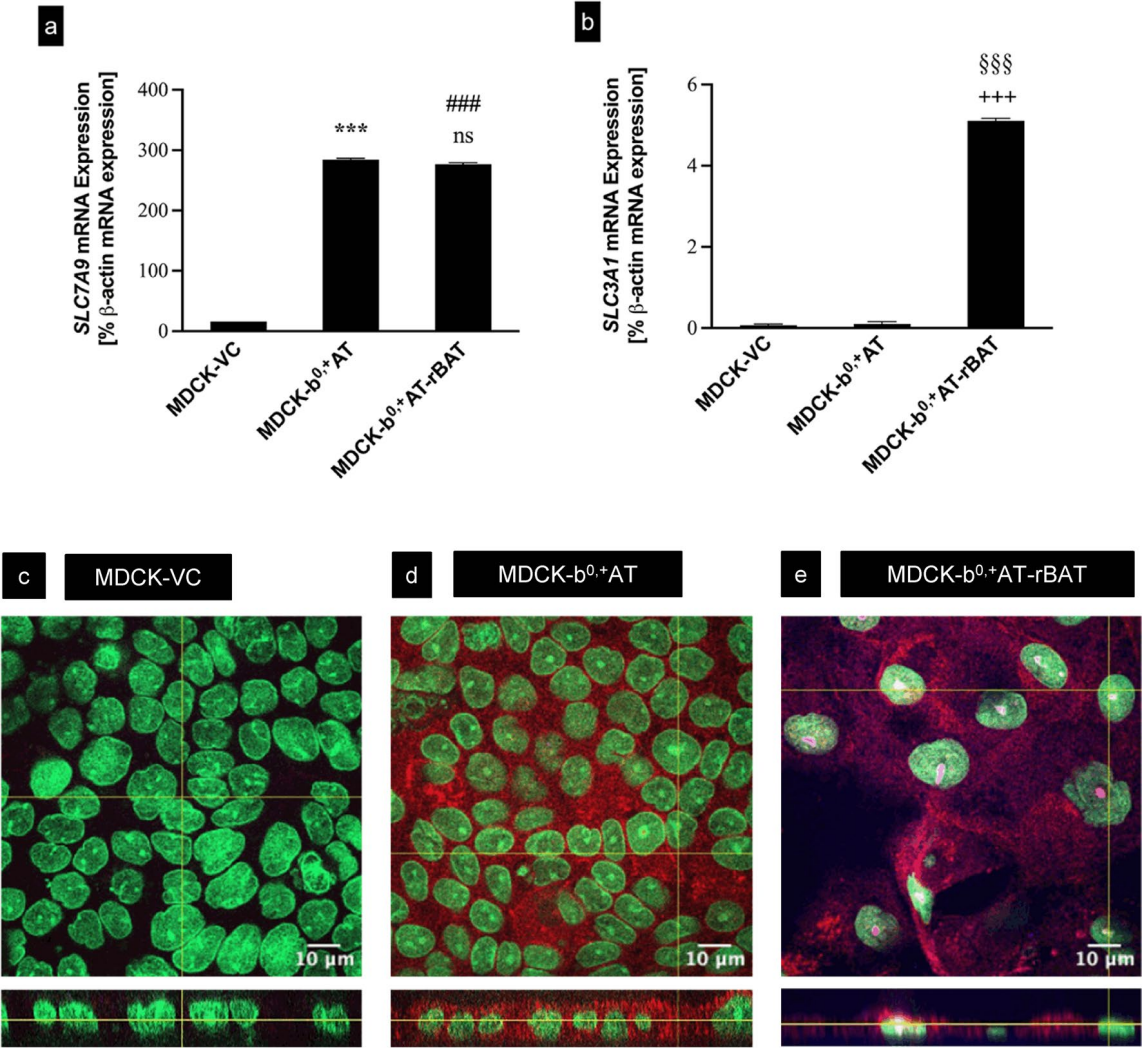
Characterization of single-transfected (MDCK-b^0,+^AT), double-transfected (MDCK-b^0,+^AT-rBAT), and the control cell line (MDCK-VC). **a** qRT-PCR analysis of *SLC7A9* (encoding human b^0,+^AT) and **b** *SLC3A1* (encoding human rBAT) mRNA expression in control cells (MDCK-VC), single-transfected MDCK-b^0,+^AT cells, and double-transfected MDCK-b^0,+^AT-rBAT cells. The mRNA expression is presented relative to the expression of the house keeping gene β-actin (*ACTB*). Data are shown as mean value ± SEM. ns (not significant) vs. MDCK-b^0,+^AT; ***p < 0.001 vs. MDCK-VC; ^###^*p* < 0.001 vs. MDCK-VC; ^+++^*p* < 0.001 vs. MDCK-VC; ^§§§^*p* < 0.001 vs. MDCK-b^0,+^AT; one-way ANOVA with Bonferroni multiple comparison test. **c**,** d**,** e** Localization of b^0,+^AT (*SLC7A9*) and rBAT (*SLC3A1*) protein. Images of confocal laser scanning microscopic analyses of MDCK-VC (**c**), the b^0,+^AT single transfectant **d**, and the b^0,+^AT-rBAT double-transfected cell line **e**. Only in cells expressing b^0,+^AT together with the rBAT subunit, the protein is localized in the apical membrane of MDCK cells. Red fluorescence indicates the expression of SLC7A9. Magenta fluorescence shows an overlay of SLC7A9 (Red) and SLC3A1 (Magenta). The nuclei are stained with sytox green (Green)

Double-transfected MDCK cell clones simultaneously expressing the subunits b^0,+^AT and rBAT were established using an MDCK-b^0,+^AT single-transfected cell line. Cell clones were selected based on mRNA expression analysis (Fig. 1). This analysis demonstrated that the expression of the *SLC7A9* mRNA encoding the b^0,+^AT subunit was comparable between single-transfected MDCK-b^0,+^AT and double-transfected MDCK-b^0,+^AT-rBAT cells (Fig. 1a). In contrast, significant *SLC3A1* mRNA expression, encoding the rBAT subunit, was detected only in the double-transfected MDCK-b^0,+^AT-rBAT cell line (Fig. 1b). Because commercially available antibodies gave no satisfactory results in immunoblot analysis, protein expression and localization of the b^0,+^AT subunit and the b^0,+^AT-rBAT heterodimer were analyzed by confocal laser scanning microscopy (Fig. 1c–e), which demonstrated that the b^0,+^AT subunit is localized intracellularly in MDCK-b^0,+^AT single transfectants (Fig. 1d). In contrast, the heterodimer was localized to the apical membrane of stably transfected MDCK-b^0,+^AT-rBAT cells (Fig. 1e).

### Uptake of L-arginine, L-homoarginine, and asymmetric dimethylarginine (ADMA)

To analyze whether L-arginine and the arginine derivatives L-homoarginine and ADMA are substrates of the heterodimeric b^0,+^AT-rBAT transporter, initial uptake experiments were performed using substrate concentrations around the endogenous plasma concentrations (which should correspond to the concentrations in the primary glomerular filtrate and thus in the proximal renal tubule). Because b^0,+^AT-rBAT was described as an uptake transporter located in the apical membrane of renal proximal tubule cells and because the localization analyses confirmed this apical localization in MDCK cells, L-arginine and both arginine derivatives were applied to the apical compartment of polarized cultured MDCK cells, and the uptake was measured after 2 min of incubation.

**Fig. 2:**
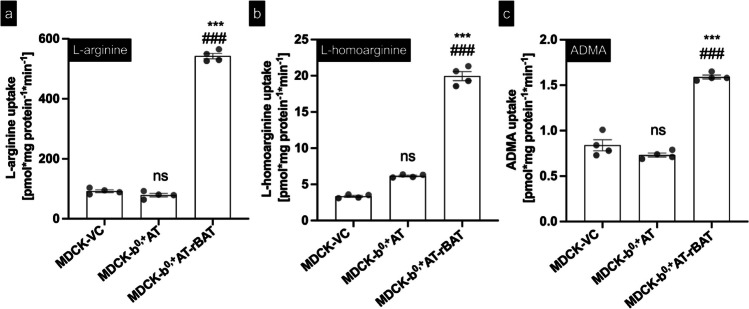
Initial uptake experiments. Labeled L-arginine (100 µM), L-homoarginine (3 µM), and ADMA (0.5 µM) were administered into the apical compartment of polarized grown MDCK-VC (black), single-transfected MDCK-b^0,+^AT (white), and double-transfected MDCK-b^0,+^AT-rBAT (grey) cells. Uptake of **a** L-arginine, **b** L-homoarginine, and **c** ADMA into the cells after 2 min is shown. Data are shown as mean value ± SEM. (*n* = 4). ns (not significant) vs. MDCK-control cells; ^***^p < 0.001 vs. MDCK-control cells; ^###^*p* < 0.001 vs. MDCK-b^0,+^AT cells; one-way ANOVA with Tukey multiple comparison test

In uptake experiments (Fig. 2), significant net uptake of L-arginine (Fig. 2a), L-homoarginine (Fig. 2b), and ADMA (Fig. 2c), relative to control cells, was only observed in MDCK cells overexpressing both subunits of the heterodimeric b^0,+^AT-rBAT transporter and not in cell lines overexpressing only b^0,+^AT. Therefore, all further experiments were conducted only with MDCK-VC cells and double-transfected MDCK-b^0,+^AT-rBAT cells.

### Characteristics of b^0,+^AT-rBAT-mediated L-arginine, L-homoarginine, and ADMA uptake

**Fig. 3:**
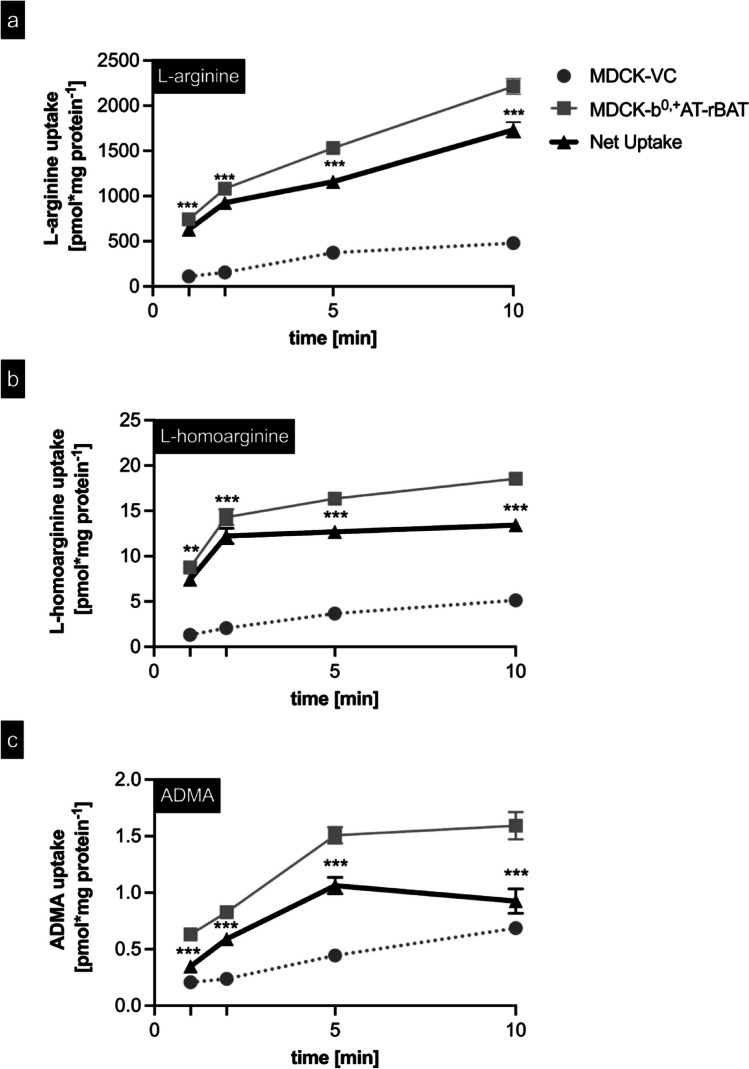
Time-dependent uptake of L-arginine, L-homoarginine, and ADMA into MDCK-VC and MDCK-b^0,+^AT-rBAT cells. **a** L-arginine 100 µM, **b** L-homoarginine 1 µM, and **c** ADMA 1 µM were administered into the apical compartment of polarized grown MDCK cells, and uptake was determined after 1, 2, 5, and 10 min. Data are shown as mean value ± SEM. (*n* = 4). **p < 0.01; ***p < 0.001; two-way ANOVA with Bonferroni multiple comparison test

To further characterize b^0,+^AT-rBAT-mediated L-arginine, L-homoarginine, and ADMA transport, time-dependent uptake experiments were performed (Fig. 3) using MDCK-VC cells and double-transfected MDCK-b^0,+^AT-rBAT cells. These results demonstrated that the uptake of all three substances was linear over the first 2 min of incubation. Therefore, 2-min substrate incubations were used for all subsequent transport experiments.

**Fig. 4:**
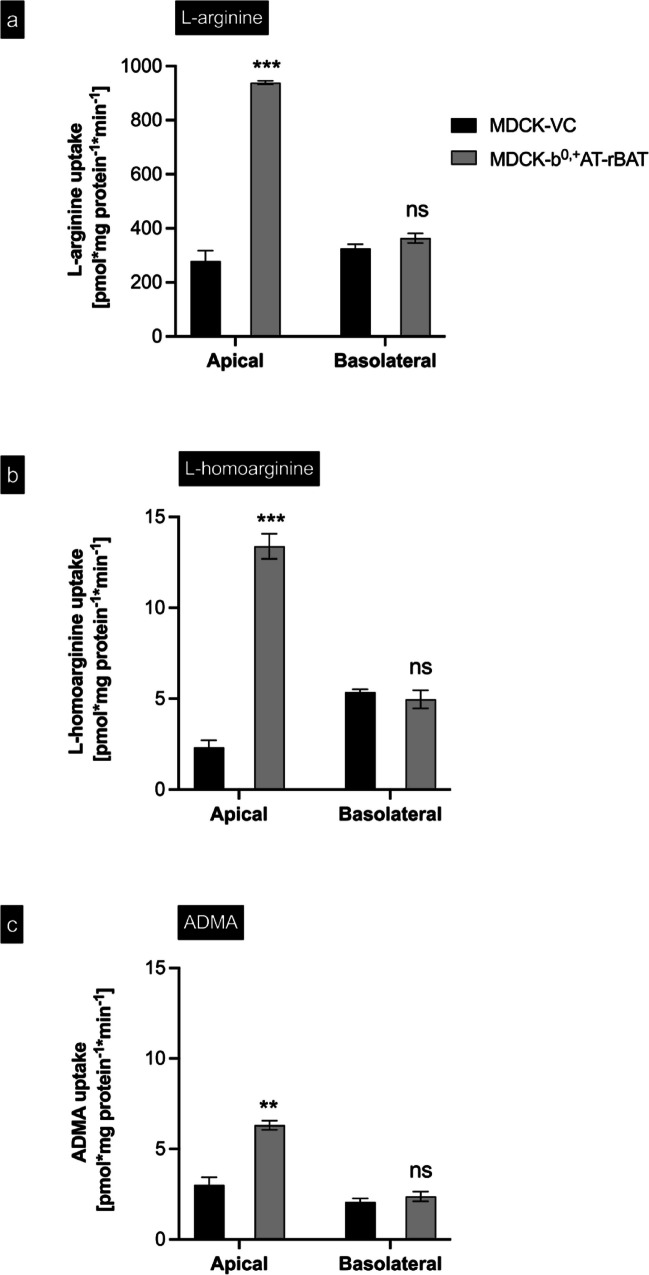
Cellular uptake across the apical or basolateral membrane of MDCK cell models. **a** L-arginine (100 µM), **b** L-homoarginine (1 µM), and **c** ADMA (1 µM) were administered into the apical or basolateral compartment of polarized grown MDCK cells, and the substrate concentration was determined after 2-min incubation time. The black bars show the uptake by MDCK-VC. The grey bars show the uptake by MDCK-b^0,+^AT-rBAT cells. Data are shown as mean value ± SEM. (*n* = 4). ns (not significant) vs. BL MDCK-VC; ***p < 0.001 vs. AP MDCK-VC; **p < 0.01 vs. AP MDCK-VC

**Fig. 5:**
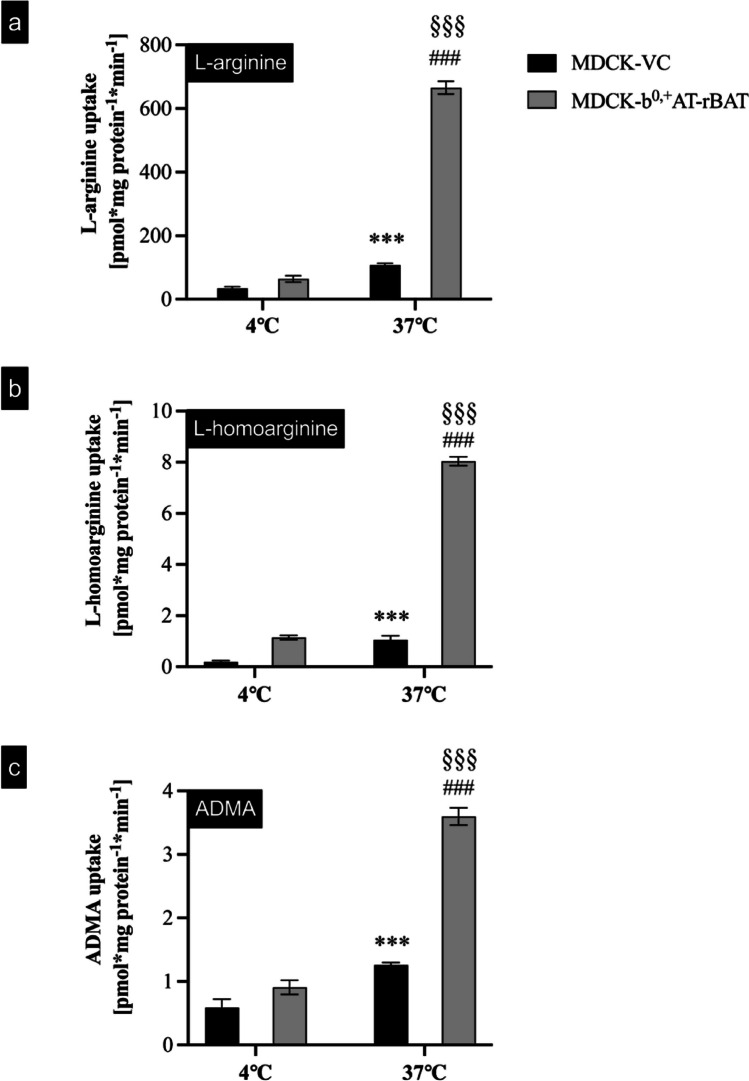
Temperature-dependent uptake of **a** L-arginine, **b** L-homoarginine, and **c** ADMA in MDCK-VC and MDCK-b^0,+^AT-rBAT cells. To test whether the transport of L-arginine, L-homoarginine, and ADMA is protein-mediated, L-arginine (100 µM), L-homoarginine (1 µM), and ADMA (1 µM) were administered into the apical compartment of the monolayers cultured at 4 °C and at 37 °C, and uptake was determined after 2 min. Data are shown as mean value ± SEM. (*n* = 4). ^***^p < 0.001 vs. 4 °C MDCK-VC; ^###^*p* < 0.001 vs. 37 °C MDCK-VC; ^§§§^*p* < 0.001 vs. 4 °C MDCK-b^0,+^AT-rBAT; two-way ANOVA with Bonferroni multiple comparison test

To discriminate between uptake across the apical and basolateral membranes, cells were incubated with 100 µM L-arginine or with 1 µM L-homoarginine or ADMA either applied to the apical or basolateral compartment of MDCK-VC or MDCK-b^0,+^AT-rBAT cells (Fig. 4). These results demonstrated significant uptake into cells only when the substrate was applied to the apical compartment. Finally, to demonstrate that the uptake is protein-dependent, temperature-dependent uptake experiments using established uptake conditions were performed at 4 °C and 37 °C (Fig. 5). These experiments showed that the b^0,+^AT-rBAT-mediated uptake of all three investigated substances was significantly reduced at 4 °C, indicating protein-dependent substrate uptake.

### Determination of the kinetic transport constants

**Fig. 6:**
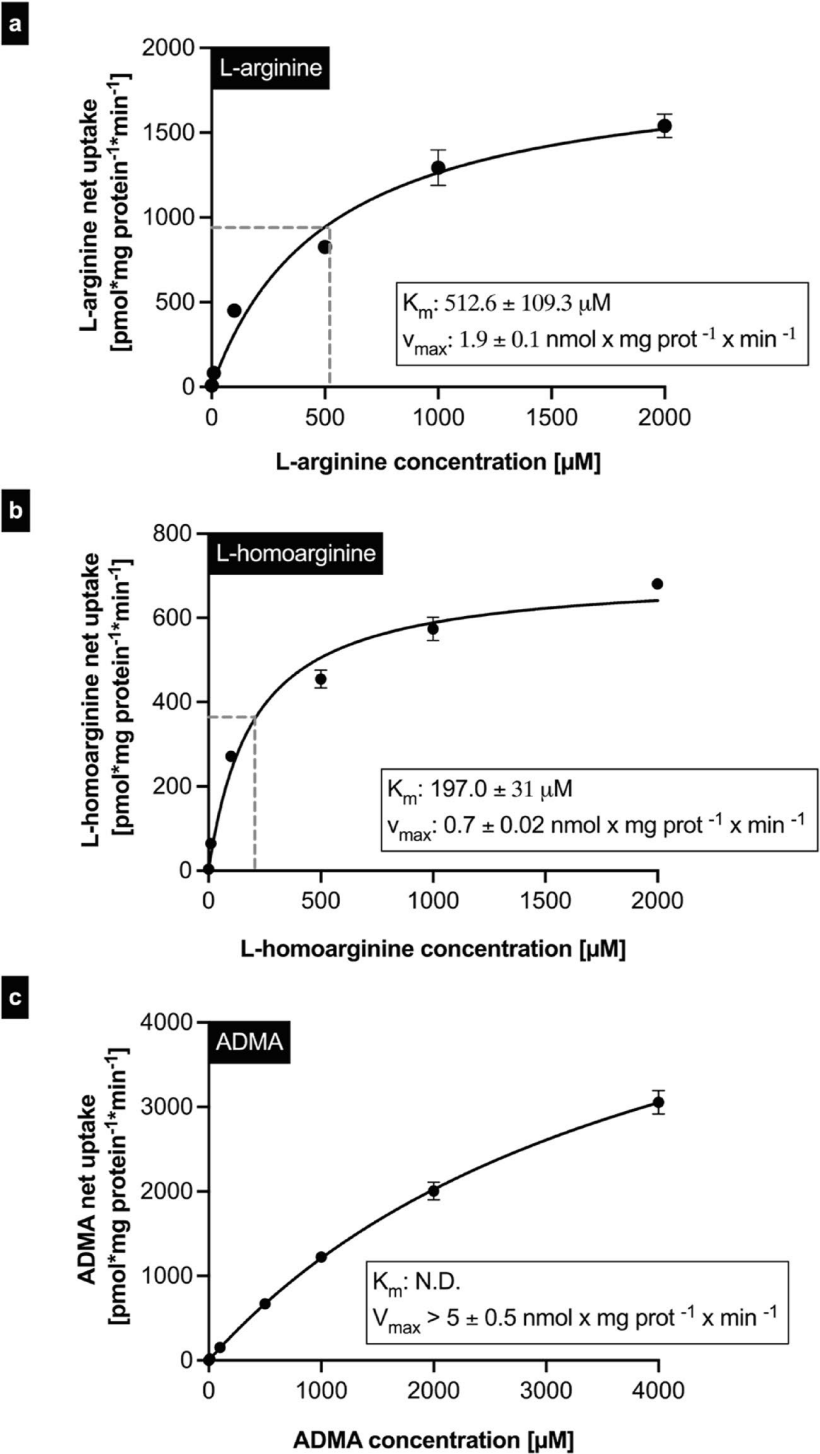
Transport kinetics of b^0,+^AT-rBAT-mediated uptake of **a** L-arginine, **b** L-homoarginine, and **c** ADMA into monolayers of MDCK-VC and MDCK-b^0,+^AT-rBAT cells. L-arginine (1; 10; 100; 500; 1,000; 2,000 µM), L-homoarginine (1; 10; 100; 500; 1,000; 2,000 µM), and ADMA (1; 10; 100; 500; 1,000; 2,000; 4,000 µM) were administered into the apical compartment of polarized grown MDCK cells, and uptake was determined after 2 min. Net uptake values were used to calculate the maximum transport velocities (*v*_max_) and of the kinetic transport constants *K*_m_ values. Data are shown as mean value ± SEM. (*n* = 4)

Concentration-dependent uptake experiments were performed using the established uptake conditions to determine the kinetic transport constant *K*_m_ values and *v*_max_ values for b^0,+^AT-rBAT-mediated uptake of L-arginine and arginine derivatives. These experiments demonstrated that for both L-arginine and L-homoarginine, the uptake was saturable, resulting in *K*_m_ values of 512.6 µM for L-arginine (Fig. 6a) and 197.0 µM for L-homoarginine (Fig. 6b). In contrast, for ADMA (Fig. 6c), no saturation in uptake could be detected, even at the highest investigated concentration of 4000 µM.

**Fig. 7:**
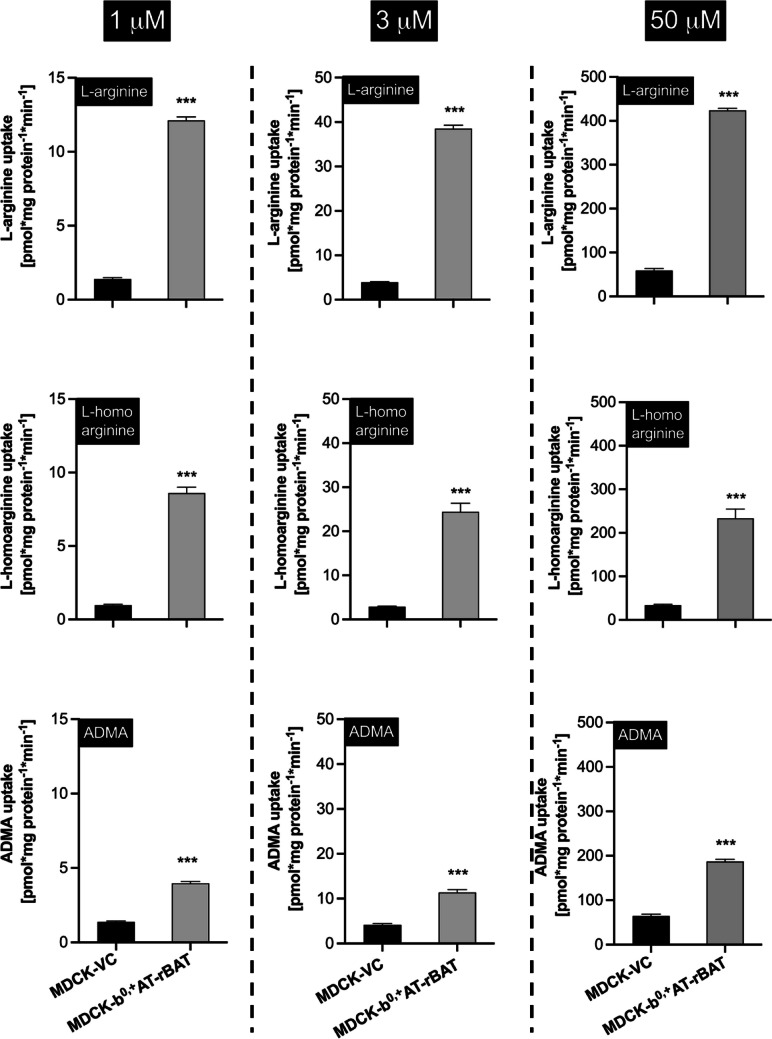
Differential handling of L-arginine and arginine derivatives by the b^0,+^AT-rBAT heterodimer. L-arginine, L-homoarginine, and ADMA were applied at concentrations of 1 µM (left graphs), 3 µM (middle graphs), and 50 µM (right graphs) into the apical compartment of polarized grown MDCK cells. Uptake into the cells after 2 min is shown. Data are shown as mean value ± SEM. (*n* = 4). ***p < 0.001 vs. MDCK-control cells; one-way ANOVA with Bonferroni multiple comparison test

This differential handling of the substrates was further investigated by comparing the uptake ratios between MDCK-VC cells and the double-transfected MDCK-b^0,+^AT-rBAT (Fig. 7) using substrate concentrations of 1 µM, 3 µM, and 50 µM. As summarized in Table 1, the uptake ratios for L-arginine and L-homoarginine varied between 6.8 and 9.8. In contrast, much lower ratios could be detected for ADMA uptake.

**Table 1:** Uptake ratios (uptake in MDCK-b^0,+^AT-rBAT cells divided by uptake into MDCK-VC cells) of L-arginine, L-homoarginine, and ADMA at the respective concentrations at an incubation time of 2 min for all substrates (quantitative data from Fig. 7)

	Average transport ratios of MDCK-b^0,+^ AT-rBAT cells relative to MDCK-VC cells		
	1 μM	3 μM	50 μM
L-arginine	8.6	9.8	7.2
L-homoarginine	8.9	8.4	6.8
ADMA	2.9	2.7	2.9

### Inhibition of L-homoarginine and ADMA uptake by L-arginine

**Fig. 8:**
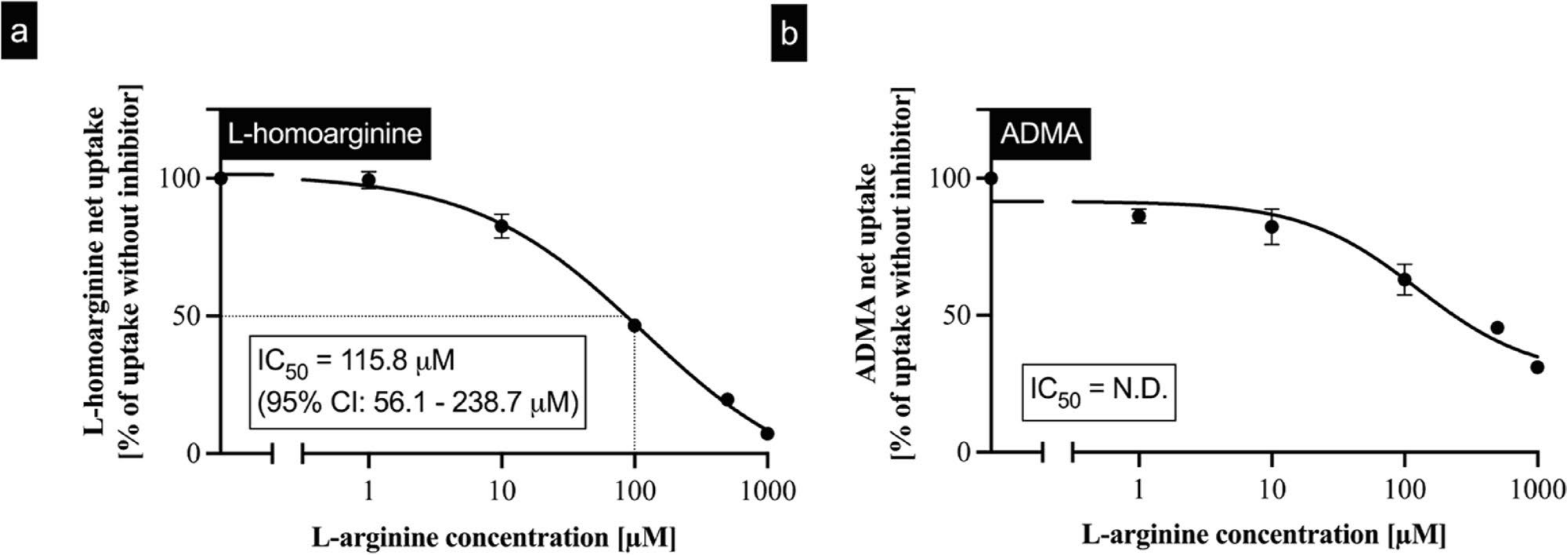
Inhibition of b^0,+^AT-rBAT-mediated uptake of **a** L-homoarginine (1 µM) and **b** ADMA (1 µM) into MDCK-VC and MDCK-b^0,+^AT-rBAT cells. L-homoarginine (1 µM) or ADMA (1 µM) was administered into the apical compartment in the absence or presence of L-arginine (1; 10; 100; 500; 1000 µM), and uptake was determined after 2 min. Data are shown as mean value ± SEM. (*n* = 4). Net uptake values were used to determine the IC_50_ value and 95% confidence interval

This selective handling of L-arginine and arginine derivatives by the b^0,+^AT-rBAT heterodimer was also assessed by inhibition experiments using L-homoarginine and ADMA as substrates and L-arginine as a potential transport inhibitor (Fig. 8). In contrast to L-homoarginine uptake, which could be efficiently inhibited by L-arginine with an IC_50_ value of 115.8 µM (Fig. 8a), b^0,+^AT-rBAT-mediated ADMA uptake could not be sufficiently inhibited (Fig. 8b). At the highest tested L-arginine concentration of 1000 µM, a residual transport of only 7% L-homoarginine could be detected. In contrast, at the same L-arginine concentration, ADMA transport was inhibited only to 31%.

### Inhibition of b^0,+^AT-rBAT-mediated uptake by L-cystine

**Fig. 9:**
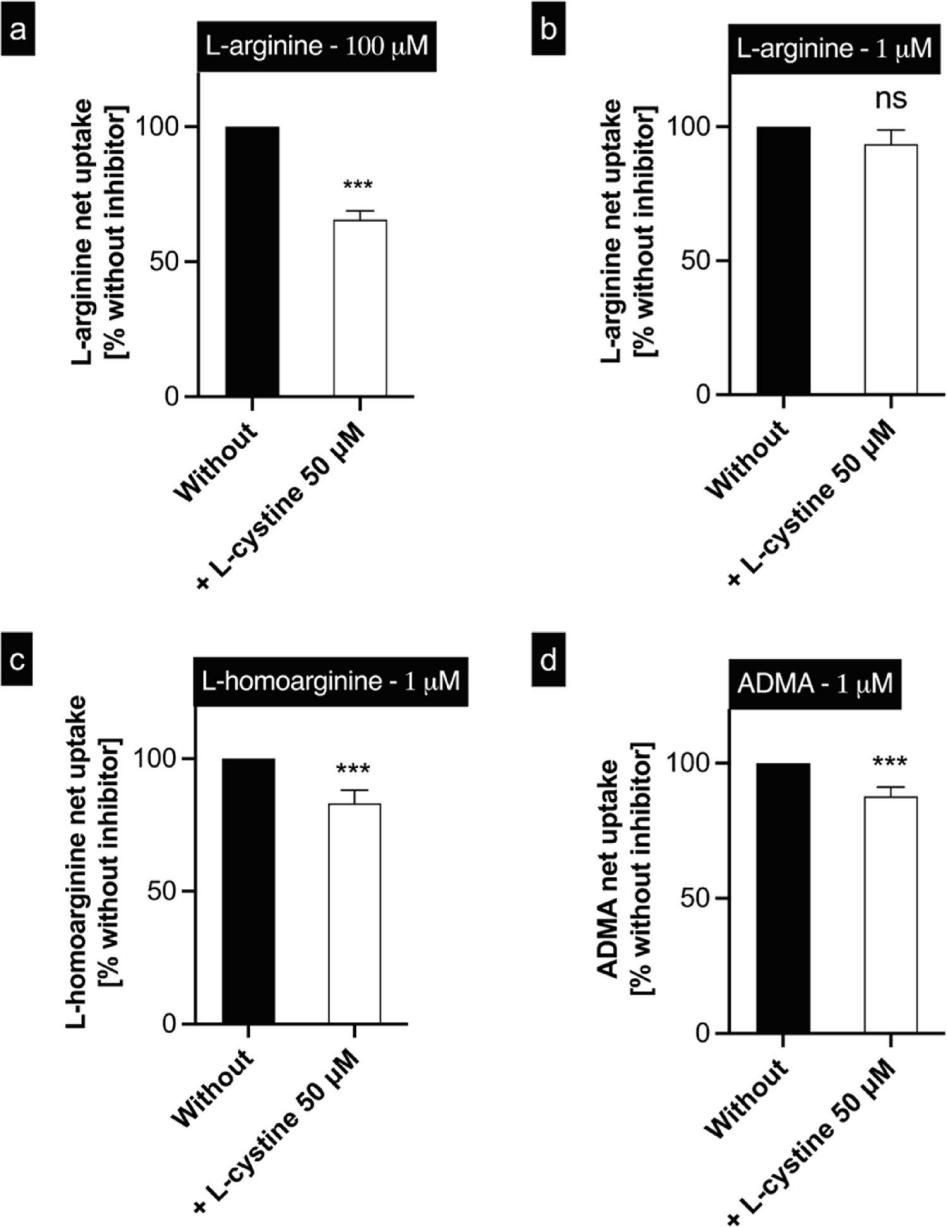
Inhibition of b^0,+^AT-rBAT-mediated uptake **a** 100 µM of L-arginine, **b** 1 µM of L-arginine, **c** 1 µM of L-homoarginine, and **d** 1 µM of ADMA by 50 µM of L-cystine. The substrate was administered into the apical compartment of polarized grown MDCK cells in the absence and presence of 50 µM L-cystine, and the uptake was determined after 2 min. Net uptake without added L-cystine (black bars) was set to 100%. Data are shown as mean value ± SEM. (*n* = 4). ns (not significant); ^***^p < 0.001 vs. without inhibitor; two-tailed test

Cystinuria is accompanied by increased concentrations of L-arginine and L-homoarginine in urine (Cox and Cameron 1974; Fjellstedt et al. 2003; Palacín 2009), suggesting that the tubular reabsorption of these substances is also impaired. Therefore, the effect of L-cystine on b^0,+^AT-rBAT-mediated L-arginine, L-homoarginine, and ADMA uptake was investigated (Fig. 9). L-arginine as a substrate was tested at low (1 µM) and high (100 µM) substrate concentrations, whereas L-homoarginine and ADMA were tested at 1 µM substrate concentration. The addition of L-cystine significantly inhibited b^0,+^AT-rBAT-mediated L-arginine uptake only at the high tested L-arginine concentration (Fig. 9a) and had no significant effect on the low L-arginine concentration (Fig. 9b). In contrast, the uptake of L-homoarginine (Fig. 9c) and ADMA (Fig. 9d) was significantly inhibited at the low tested concentrations of 1 µM.

## Discussion

Using stably transfected MDCK cells and radiolabeled substrates, we characterized b^0,+^AT-rBAT-mediated transport of L-arginine and its derivatives L-homoarginine and ADMA. All three substances were found to be b^0,+^AT-rBAT substrates but differed in their transport kinetics. Significant net uptake into cells was only observed when the substrate was applied to the apical compartment, verifying b^0,+^AT-rBAT-mediated substrate uptake across the apical membrane in the experimental setting, corresponding to the luminal (urine-facing) membrane of proximal tubule epithelial cells.

In direct comparisons of the three substates, the uptake ratios (Table 1) and transport rates (Figs. 6 and 8) of L-arginine and L-homoarginine were consistently higher than those of ADMA. Furthermore, our data reveals that L-cystine, a long known substrate of b^0,+^AT-rBAT, inhibits L-arginine, L-homoarginine, and ADMA uptake at physiological concentrations (Fig. 9). Taken together, the differences in transport properties, as well as the inhibition experiments, all point to a differential (i.e., partially selective) handling of the arginine derivatives by b^0,+^AT-rBAT, with a relative preference of L-arginine > L-homoarginine >  > ADMA. While this selective preference is compatible with the observed differences in the renal clearance of the three substrates (Atzler et al. 2011; Banjarnahor et al. 2020; Brosnan and Brosnan 2004; May et al. 2015; Tizianello et al. 1980; Torremans et al. 2003), the differential handling of L-arginine and its derivatives by the kidneys likely results from a combination of several factors rather than a single factor.

Further insights regarding the b^0,+^AT-rBAT transport dynamics might also be gained from assessment of competition of cationic with neutral amino acids, but the interpretation of these data may be difficult. Leucine uptake by b^0,+^AT-rBAT has been reported in OK cells (Mora et al. 1996) with a K_m_ value of 175 μM. However b^0,+^AT-BAT acts as an exchanger, which has been explicitly described by Yan et al. The authors explain that cystine is transported when one of two ends of cystine binds to the lower end of pocket 2, which comprises Asp233, Trp235, and Tyr386 (Yan et al. 2020). The same binding sites also accommodate the polar moiety of the side chain of the cationic amino acids (Yan et al. 2020). These results indicated that cystine and cationic amino acids share common binding sites at the transporter. In contrast, neutral amino acids appear to show a different binding pattern.

Other transport proteins can mediate L-arginine uptake and its derivatives from (or release into) the urine. It is possible that not all transport proteins capable of mediating the uptake or release of L-arginine and its derivatives by tubular cells have yet been identified (Banjarnahor et al. 2020). Therefore, there could be yet unknown but even more selective transport processes for the arginine derivatives. These may offer alternative explanations for some of our observations such as the apparent incomplete inhibition of b^0,+^AT-rBAT-mediated uptake of ADMA. Which could also be explained by the residual presence, or even upregulation, of other transport proteins in our cell model that transport ADMA, but not L-homoarginine (for which the transport could be almost completely inhibited by high concentrations of L-arginine). However, considering the hyperargininuria and hyperhomoargininuria observed in patients with deficient b^0,+^AT-rBAT function (Cox and Cameron 1974; Fjellstedt et al. 2003; Palacín 2009), b^0,+^AT-rBAT can be expected to play a major role in the different renal handling of these substances. In healthy adults, the endogenous clearance of lysine and arginine is less than 1 mL per minute, and tubular reabsorption is nearly complete (> 99%). However, in patients with cystinuria, the endogenous clearance of lysine is approximately 55 mL per minute (Doolan et al. 1957). Comparable results were seen for arginine in cystinuria patients, with an average renal clearance of 60 mL per minute (Crawhall et al. 1967). L-cystine displayed distinct effects on the uptake of L-arginine, L-homoarginine, and ADMA by the b^0,+^AT-rBAT transporter, indicating that these interactions are concentration-dependent and substrate-specific.

Our observation that L-cystine only partly inhibits the uptake of L-arginine at physiological concentrations complements a previous report by Wu et al., who demonstrated that 50 µM L-cystine resulted in a 40% inhibition of L-arginine uptake by a genetically modified (tagged) version of the b^0,+^AT-rBAT heterodimer (Wu et al. 2020). The somewhat counterintuitive observation that L-arginine transport at higher concentrations (100 µM) is inhibited by 50 µM of L-cystine but not at lower concentrations (1 µM) could be explained by different substrate affinities and allosteric effects dominating the interaction at lower concentrations while at higher concentrations of L-arginine the classic competitive interaction prevails, as described earlier for other substrates and SLC-transport proteins (Kindla et al. 2011).

Given the importance of L-cystine and cationic amino acids in physiological pathways, it is expected that b^0,+^AT-rBAT possesses a unique binding site specifically for these compounds (Wu et al. 2020). The observed IC_50_ of 115.8 µM for inhibiting L-homoarginine uptake by L-arginine is within the physiological range of L-arginine in plasma and thus may become potentially relevant. However, patients with lysinuric protein intolerance (LPI), which is caused by mutations in the *SLC7A7* gene, also show increased urinary L-arginine and L-homoarginine excretion (Kato et al. 1989; Torrents et al. 1999). *SLC7A7* encodes y^+^LAT1, a transport protein expressed in the basolateral membrane of renal tubular cells responsible, among others, for the cellular release of L-arginine, L-homoarginine, and ADMA (Closs et al. 2012). Making this transporter a possible alternative contributing factor to be considered.

Furthermore, ADMA can be taken up from the blood and excreted into the urine by various other transport proteins. Among others, OATP4C1 (encoded by the *SLCO4C1* gene) is expressed in the basolateral membrane of tubular cells. OATP4C1 has already been shown to mediate the cellular uptake and release of L-arginine, L-homoarginine, and ADMA from cells (Taghikhani et al. 2019). Conversely, release into the urine can be mediated by proteins in the apical membrane of proximal tubule cells, such as MATE1 (gene symbol *SLC47A1*) (Strobel et al. 2013). However, the relative contribution of MATE1 and other transport proteins to the overall renal excretion of ADMA across the luminal membrane of proximal tubule cells remains to be investigated.

Being substrates of transport proteins aside, L-arginine, L-homoarginine, and ADMA are also substrates and products of different sets of enzymes expressed in the kidney. Thus, it is conceivable that the combined net effect of synthesis and metabolism by these enzymes may also contribute to the observed differential renal handling of arginine derivatives. In this respect, it has previously been suggested that a combination of renal filtration, tubular secretion, tubular reabsorption, and intracellular enzymatic degradation in proximal tubule cells is crucial for eliminating ADMA from the body (Nijveldt et al. 2002). To further clarify this issue, assessing the uptake of symmetric dimethylarginine (SDMA), which has a renal excretion similar to ADMA but undergoes somewhat limited metabolism (compared to ADMA), may help to further clarify the role of metabolism.

## Conclusion

Taken together, the data of the present study indicate a preference (i.e., greater affinity) of b^0,+^AT-rBAT towards L-arginine and L-homoarginine, as compared to the uremic toxin ADMA. This aligns with the observation that L-arginine and L-homoarginine are almost completely retained in the kidneys, while ADMA is excreted in urine. The observed differential handling of arginine derivatives by the b^0,+^AT-rBAT heterodimeric transport protein may open new mechanistic and possible therapeutic approaches to address pathologies associated with relative excess or deficiencies of arginine derivatives such as ADMA, SDMA, and L-homoarginine.

## Data Availability

Raw data supporting the findings of this study are available from the corresponding author [RM] on reasonable request.
